# Lobectomy vs Total Thyroidectomy With Ipsilateral Lateral Neck Dissection for N1b Intermediate-Risk Papillary Thyroid Carcinoma

**DOI:** 10.1001/jamaoto.2024.3860

**Published:** 2024-11-27

**Authors:** Yoshiyuki Saito, Kenichi Matsuzu, Amr H. Abdelhamid Ahmed, Kosuke Inoue, Hiroshi Shibuya, Ai Matsui, Yoko Kuga, Reiko Ono, Kana Yoshioka, Chie Masaki, Junko Akaishi, Kiyomi Y. Hames, Ritsuko Okamura, Chisato Tomoda, Akifumi Suzuki, Wataru Kitagawa, Mitsuji Nagahama, Kiminori Sugino, Hiroshi Takami, Gregory W. Randolph, Koichi Ito

**Affiliations:** 1 Department of Surgery, Ito Hospital, Tokyo, Japan; 2 Division of Thyroid and Parathyroid Endocrine Surgery, Department of Otolaryngology–Head and Neck Surgery, Massachusetts Eye and Ear Infirmary, Harvard Medical School, Boston; 3 Department of Social Epidemiology, Graduate School of Medicine and School of Public Health/Hakubi Center, Kyoto University, Kyoto, Japan; 4 Shibuya Thyroid Clinic, Tokyo, Japan; 5 Department of Surgery, Massachusetts General Hospital, Harvard Medical School, Boston

## Abstract

**Importance:**

The management of papillary thyroid carcinoma (PTC), particularly in cases with clinically apparent lateral neck lymph node metastasis (cN1b), remains an area of debate. The surgical options for PTC, including total thyroidectomy and lobectomy, have distinct impacts on patients’ outcomes and quality of life.

**Objective:**

To compare survival and recurrence outcomes between patients who underwent a lobectomy plus ipsilateral lateral neck dissection (LND) and those who underwent a total thyroidectomy plus ipsilateral LND for intermediate-risk cN1b PTC with both primary tumors and lymph node metastases in the ipsilateral neck region.

**Design, Setting, and Participants:**

This retrospective cohort study was conducted at Ito Hospital, Tokyo, Japan. Patients who underwent surgery for PTC between January 2005 and December 2012 were included, and those with high-risk PTCs and concurrent other thyroid cancers were excluded. Data were analyzed from April to August 2024.

**Exposures:**

Lobectomy plus LND vs total thyroidectomy plus LND.

**Main Outcomes and Measures:**

An inverse probability of treatment weighting (IPTW)–adjusted Kaplan-Meier analysis and a Cox proportional hazards regression analysis were performed to compare the patients’ overall survival, recurrence-free survival (RFS), and modified RFS (which considered the potential need for a future contralateral lobectomy).

**Results:**

Of 401 included patients, 317 (79.1%) were female, and the median (IQR) age was 47 (36-59) years. A total of 157 patients underwent lobectomy plus ipsilateral LND and 244 underwent total thyroidectomy plus ipsilateral LND. The median (IQR) follow-up time was 13.0 (11.2-15.0) years. The IPTW-adjusted overall survival rates at 5, 10, and 15 years were 98.0% (95% CI, 93.9-99.3), 97.5% (95% CI, 93.2-99.1), and 96.8% (95% CI, 92.2-98.7), respectively, for the lobectomy group vs 99.4% (95% CI, 97.0-99.9), 97.4% (95% CI, 94.4-98.8), and 96.9% (95% CI, 93.3-98.5), respectively, for the total thyroidectomy group (hazard ratio [HR], 1.10; 95% CI, 0.35-3.47). The IPTW-adjusted RFS rates at 5, 10, and 15 years were 93.8% (95% CI, 88.5-96.7), 88.4% (95% CI, 82.0-92.6), and 84.1% (95% CI, 76.8-89.3), respectively, for the lobectomy group vs 95.4% (95% CI, 91.8-97.4), 92.9% (95% CI, 88.8-95.5), and 87.8% (95% CI, 80.8-92.4), respectively, for the total thyroidectomy group (HR, 1.41; 95% CI, 0.79-2.54). The IPTW-adjusted modified RFS rates at 5, 10, and 15 years were 96.7% (95% CI, 92.2-98.6), 93.8% (95% CI, 88.5-96.7), and 88.9% (95% CI, 82.4-93.1), respectively, for the lobectomy group vs 95.4% (95% CI, 91.8-97.4), 92.9% (95% CI, 88.8-95.5), and 87.8% (95% CI, 80.8-92.4), respectively, for the total thyroidectomy group (HR, 0.93; 95% CI, 0.49-1.76).

**Conclusions and Relevance:**

In this study, for these selected intermediate-risk cN1b PTC cases, total thyroidectomy and lobectomy provided comparable outcomes in terms of prognosis and recurrence. These data may help inform future guideline revisions and support joint decision-making between patients and their clinicians.

## Key Points

**Question:**

What are the comparative outcomes of lobectomy plus ipsilateral lateral neck dissection (LND) and total thyroidectomy plus ipsilateral LND for intermediate-risk cN1b papillary thyroid carcinoma (PTC)?

**Findings:**

In this cohort study including 401 patients, the postinverse probability of a treatment weighting analysis showed no clinically meaningful differences in overall survival, recurrence-free survival, or modified recurrence-free survival between the 2 surgical approaches.

**Meaning:**

In this study, for patients with intermediate-risk cN1b PTC limited to the unilateral thyroid lobe and ipsilateral lateral neck lymph nodes, lobectomy plus ipsilateral LND and total thyroidectomy plus ipsilateral LND yielded similar outcomes in terms of overall survival and recurrence.

## Teaser

This cohort study compares survival and recurrence outcomes between patients who underwent a lobectomy plus ipsilateral lateral neck dissection (LND) and those who underwent a total thyroidectomy plus ipsilateral LND for intermediate-risk cN1b papillary thyroid carcinoma with both primary tumors and lymph node metastases in the ipsilateral neck region.

## Introduction

Papillary thyroid carcinoma (PTC) is the most common type of thyroid cancer, and its management has been a subject of ongoing debate among endocrinologists, surgeons, and oncologists. The landscape of surgical management for PTC has witnessed notable shifts. Traditionally, extensive procedures, such as prophylactic central neck dissection (CND) and lateral neck dissection (LND), were common. However, guidelines published in the last decade no longer recommend routine prophylactic LND,^1^ and prophylactic CND is now considered unnecessary for low-risk PTC, as it may not benefit all patients.^2,3,4^ The choice between total thyroidectomy and thyroid lobectomy has also evolved, with lobectomy increasingly considered a viable option in select cases. In other words, treatment decisions for patients with PTC will be assessed based on whether there is an advantage to prophylactic contralateral lobectomy. It is important to distinguish this from completion lobectomy, which is defined as the surgical removal of the contralateral remnant thyroid tissue following an initial procedure that is less than a total thyroidectomy. In contrast, prophylactic contralateral lobectomy refers to the simultaneous removal of the contralateral thyroid lobe during the initial surgery, aiming to prevent potential future recurrence.^5^

The choice between lobectomy and total thyroidectomy is a complex decision, as it directly impacts the patient’s quality of life, potential postoperative complications, and long-term oncological outcomes. Lobectomy, a more conservative approach, aims to preserve thyroid function and reduce the risk of complications, such as hypothyroidism. A total thyroidectomy is often thought to provide more comprehensive disease control, with the additional benefit of facilitating radioactive iodine (RAI) treatment, but it results in the necessity of lifelong thyroid hormone therapy. Particularly in East Asian regions, including Japan, there have been several reports advocating for the choice of lobectomy.^6,7,8^ The guidelines of the American Thyroid Association (ATA) and the US National Comprehensive Cancer Network now recognize that a prophylactic contralateral lobectomy may not be universally necessary, particularly in cases of low-risk PTC.^4,9^ The assessment of individual patients’ circumstances and risks has thus become increasingly important.

PTC represents a heterogeneous spectrum of clinical presentations, and the classification of patients into risk groups (low, intermediate, and high) has played a pivotal role in guiding treatment decisions. The ATA guidelines^4^ recommend RAI therapy for high-risk PTC cases, such as those with gross extensive extrathyroidal extension and/or distant metastases, where clear benefits in terms of prognosis have been observed. Conversely, the ATA guidelines discourage the routine use of RAI therapy in low-risk PTC cases, where the potential benefits are not as evident. The role of RAI therapy in intermediate-risk PTC remains uncertain.^10,11,12^ The ATA guidelines prompted us to question whether total thyroidectomy and RAI remnant ablation are required to facilitate the follow-up of patients with intermediate-risk PTC.

Among the various factors influencing treatment decisions for PTC, the extent of surgical intervention (ie, total thyroidectomy or lobectomy) remains a critical consideration, particularly in the cases of patients with intermediate-risk PTC with clinically apparent lateral neck lymph node metastasis (cN1b).^13^ The National Comprehensive Cancer Network guidelines^9,14^ recommend a total thyroidectomy for cases of intermediate-risk PTC with clinically apparent lateral neck lymph node metastasis, whereas the corresponding Japanese guidelines^15^ present both total thyroidectomy and thyroid lobectomy as options for such cases.

**Figure 1.**
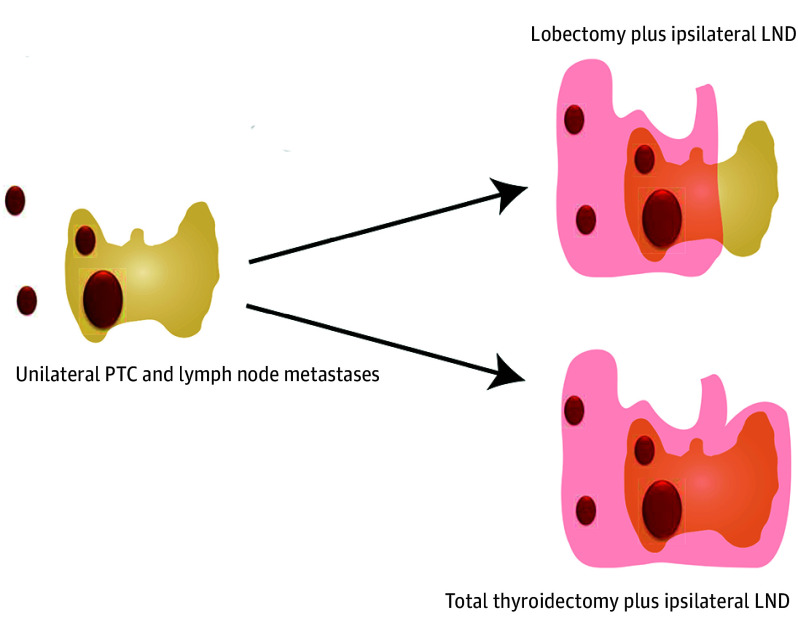
Surgical Options for Papillary Thyroid Carcinoma (PTC) Limited to the Unilateral Thyroid Lobe and Ipsilateral Lateral Neck Lymph Nodes LND indicates lateral neck dissection.

Our hospital specializes in the treatment of patients with thyroid diseases and has long collected clinical data on many PTC cases.^7,16^ The primary objective of the present study was to compare the survival and recurrence outcomes between patients who underwent a lobectomy and those who underwent a total thyroidectomy for intermediate-risk cN1b PTC with confirmed primary tumors and lymph node metastases in the ipsilateral neck region only. By examining these 2 surgical approaches, we seek to contribute to the ongoing discourse surrounding the management of intermediate-risk PTC and to provide valuable insights into the selection of the most appropriate surgical strategy for these patients (Figure 1).

## Methods

### Study Design and Patients

We analyzed cases of patients who underwent surgery for PTC or suspected PTC between January 2005 and December 2012 at our institution. During this period, 24 surgeons performed procedures for patients with PTC. Patients were eligible if they had confirmed cN1b status with PTC limited to a unilateral thyroid lobe and ipsilateral lateral neck lymph nodes, based on a preoperative examination. We compared 2 surgical approaches: lobectomy plus ipsilateral LND and total thyroidectomy plus ipsilateral LND. We excluded patients with PTC exhibiting high-risk factors as defined in the ATA guidelines, including (1) distant metastases, (2) any metastatic lymph node 3 cm or larger in largest dimension, (3) T4 disease, or (4) incomplete tumor resection. Patients with final pathology indicating non-PTC or concurrent other thyroid cancers were also excluded. Approval to conduct this clinical study was granted by the Ito Hospital Institutional Review Board, and the study was performed in accord with the Strengthening the Reporting of Observational Studies in Epidemiology (STROBE) reporting guideline. The approach of opt-out consent was used for our retrospective analysis. Retrospective deidentified data were used, and informed consent was waived by the institutional review board.

Staged thyroidectomy was not performed because intraoperative neuromonitoring (IONM) was not used during the study period. Patients who underwent a 2-stage total thyroidectomy were thus not included in the study. In the total thyroidectomy group, bilateral CND was performed, whereas in the lobectomy group, only the affected side was dissected for all patients. The choice between total thyroidectomy plus ipsilateral LND and lobectomy plus ipsilateral LND was not standardized.

### Study Variables

Data were prospectively collected from the institutional surgical database and included age, sex, preoperative TNM classification, preoperative and intraoperative extrathyroidal extension findings, surgical procedures, completeness of tumor resection, and various pathological findings. Preoperative tumor size data were based on ultrasound measurements obtained before surgery.

The primary end point was overall survival (OS), measured from the initial surgery until death or censorship at the last follow-up. The secondary end point was recurrence-free survival (RFS). Instead of prophylactic lymph node dissection at the time of an initial surgery, it has become acceptable to perform therapeutic lymph node dissections when structural lymph node metastases are detected. As a result, the number of individuals undergoing unnecessary prophylactic lymph node dissections has been reduced. We thus introduce the concept of modified RFS in this study. Modified RFS excludes recurrences limited to areas that could have been resected if the initial surgery had been a total thyroidectomy (ie, remaining thyroid or a contralateral level VI lymph node). The use of RFS tailored to particular outcomes is a recognized and valuable approach in clinical research.^17,18,19^ Details of modified RFS used herein are described in the eMethods in Supplement 1.

### Statistical Analysis

To account for potential differences in patient backgrounds between the 2 patient groups (lobectomy plus ipsilateral LND vs total thyroidectomy plus ipsilateral LND), we used inverse probability of treatment weighting (IPTW) to adjust for patient age and sex, tumor size, multifocal tumor status, metastasis to level VI lymph nodes, the largest metastatic lymph node size, and the number of lymph node metastases at levels II, III, and IV. Absolute values of the standardized mean differences less than 10% were considered a successful balance for each covariate between the 2 groups.^20^ We conducted an IPTW-adjusted Kaplan-Meier analysis and a Cox proportional hazards regression analysis to compare the OS, RFS, and modified RFS rates between the 2 groups. The statistical analyses were conducted using Stata software version 15.0 (StataCorp) or R version 4.2.2 (The R Foundation). Data were analyzed from April to August 2024.

## Results

### Study Population

**Figure 2.**
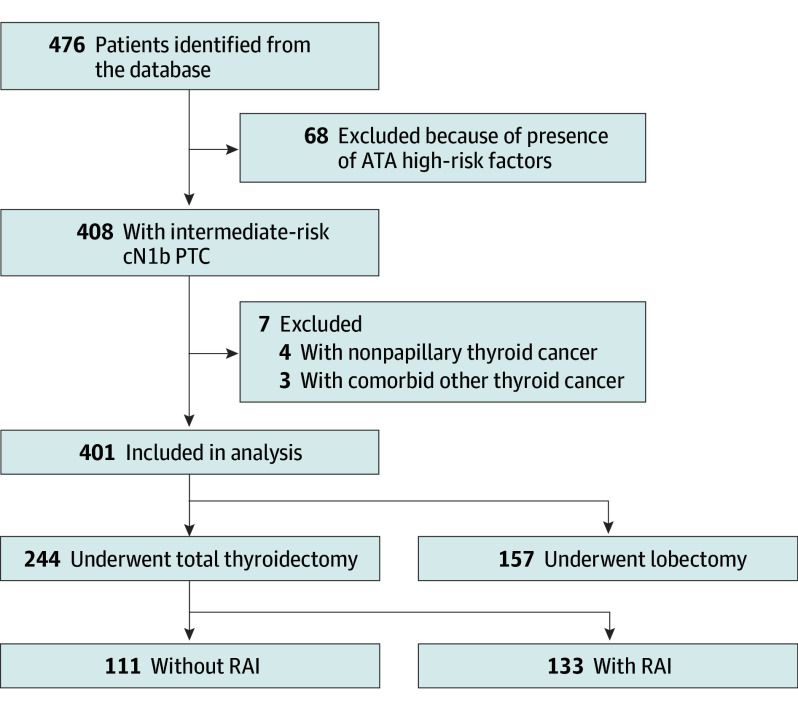
Flowchart of Patient Enrollment in the Study American Thyroid Association (ATA) high-risk factors include papillary thyroid carcinoma (PTC) with (1) distant metastases, (2) any metastatic lymph node 3 cm or larger in largest dimension, (3) T4 tumor stage, or (4) incomplete tumor resection. cN1b indicates clinically apparent lateral neck lymph node metastasis; RAI, radioactive iodine.

Figure 2 presents the patient flowchart. A total of 476 candidates were identified from the database. Among these, 68 patients were excluded due to the presence of ATA high-risk factors, and 7 patients were excluded based on pathological criteria: 4 for nonpapillary thyroid cancer and 3 for comorbid other thyroid cancers. Of 401 included patients, 317 (79.1%) were female, and the median (IQR) age was 47 (36-59) years. A total of 157 underwent a lobectomy plus ipsilateral LND and 244 underwent a total thyroidectomy plus ipsilateral LND.

Table 1 summarizes the clinicopathologic characteristics of the enrolled patients before and after the IPTW adjustment. Before IPTW, the patients who underwent a lobectomy differed from those who underwent a total thyroidectomy in several aspects. Patients undergoing lobectomy tended to be younger than those undergoing total thyroidectomy (mean [SD] age, 46.6 [15.6] years vs 48.6 [14.2] years) and had a higher proportion of male sex (41 of 157 [26%] vs 43 of 244 [18%]). They also had lower rates of unilateral multifocal tumors (32 [20%] vs 77 [32%]) and preoperative confirmed metastasis to level VI lymph nodes (69 [44%] vs 123 [50%]), and they had smaller-sized metastatic lymph nodes (mean [SD] largest diameter, 13.5 [5.7] mm vs 15.2 [5.9] mm). Patients undergoing lobectomy also had fewer pathologically confirmed lymph node metastases in the lateral neck area (mean [SD] number of metastatic lymph nodes, 3.9 [2.9] vs 4.9 [3.0]).

**Table 1.** Baseline Characteristics of the Study Patients Before and After the Inverse Probability of Treatment Weighting

Characteristic	Unweighted study population			Weighted study population		
	Lobectomy (n = 157)	Total thyroidectomy (n = 244)	Standardized difference, %	Lobectomy	Total thyroidectomy	Standardized difference, %
Preoperative characteristics						
Age, y						
Mean (SD)	46.6 (15.6)	48.6 (14.2)	−13.1	47.9	48.1	−1.3
Median (IQR)	45.0 (35.0-58.0)	48.5 (39.0-60.0)		NA	NA	
Sex						
Male	41 (26)	43 (18)	−20.6	22	21	−1.8
Female	116 (74)	201 (82)		78	79	
Tumor size, mm						
Mean (SD)	21.9 (12.3)	22.0 (14.3)	−0.4	22.4	22.2	1.6
Median (IQR)	18.6 (13.5-28.4)	18.6 (11.4-30.2)		NA	NA	
Multifocal tumor^a^	32 (20)	77 (32)	−25.6	26	27	−3.0
Metastasis to level VI lymph nodes	69 (44)	123 (50)	−12.9	47	48	−1.6
Largest metastatic lymph node size, mm						
Mean (SD)	13.5 (5.7)	15.2 (5.9)	−28.7	14.4	14.6	−2.8
Median (IQR)	12.3 (9.2-17.2)	14.4 (10.7-19.0)		NA	NA	
Pathological finding						
Lymph node metastases contained in levels II, III, and IV						
Mean (SD)	3.9 (2.9)	4.9 (3.0)	−34.2	4.4	4.5	−4.5
Median (IQR)	3.0 (2.0-5.0)	4.0 (2.3-7.0)		NA	NA	

Abbreviation: NA, not applicable.

^a^ All multifocal tumors were unilateral because patients with bilateral multifocal tumors were not enrolled in this study.

The difference in tumor size was very small between the lobectomy and total thyroidectomy groups (mean [SD] size, 21.9 [12.3] mm vs 22.0 [14.3] mm). After the IPTW adjustment, all standardized differences were less than 10%, indicating an adequate balance of clinicopathologic characteristics between the 2 treatment groups. Additional details about the pathological and intraoperative findings are provided in the eResults in Supplement 1. The IPTW-adjusted rate of RAI remnant ablation after surgery was 0% in the patients who underwent a lobectomy and 53.8% (131.2 of 244.1) in the patients who underwent a total thyroidectomy. There was a very small and clinically insignificant difference in OS or RFS between the patients who underwent a total thyroidectomy with or without RAI, suggesting that the impact of RAI in this intermediate-risk series was limited; we conducted subsequent analyses with this consideration (eFigure 1 in Supplement 1). In terms of the complications associated with total thyroidectomy, the rates of postoperative permanent hypoparathyroidism and transient bilateral recurrent laryngeal nerve (RLN) paralysis were 9.4% (23 of 244) and 0.8% (2 of 244), respectively, in the total thyroidectomy group, while no patients in the lobectomy group had these complications (eTable in Supplement 1).

### Determinant Analysis

We performed a multivariable logistic regression analysis to identify any determinants of a patient being assigned a lobectomy vs a total thyroidectomy, and the results are presented in Table 2. Regarding this choice of surgery, the factors favoring a lobectomy plus ipsilateral LND included male sex and the presence of a unifocal tumor, whereas larger metastatic lymph nodes and a higher number of lymph node metastases in the lateral neck area were associated with a preference for total thyroidectomy. The patient age, primary tumor size, and the presence of lymph node metastases in the central neck area did not significantly impact the choice of surgery.

**Table 2.** Multiple Logistic Regression Model Determining the Choice of Lobectomy Plus Ipsilateral Lateral Neck Dissection vs Total Thyroidectomy Plus Ipsilateral Lateral Neck Dissection

Variable	OR (95% CI)	*P* value
Preoperative characteristics		
Age	0.98 (0.97-1.00)	.06
Sex		
Male	1 [Reference]	NA
Female	0.50 (0.30-0.84)	.009
Tumor size, mm	1.00 (0.99-1.02)	.90
Multifocal tumor	0.57 (0.34-0.94)	.03
Metastasis to level VI lymph nodes	0.88 (0.56-1.36)	.56
Largest metastatic lymph node size, mm	0.95 (0.91-0.98)	.005
Pathological finding		
Lymph node metastases contained in levels II, III, and IV	0.89 (0.83-0.96)	.003

Abbreviations: NA, not applicable; OR, odds ratio.

### Survival Analysis

**Figure 3.**
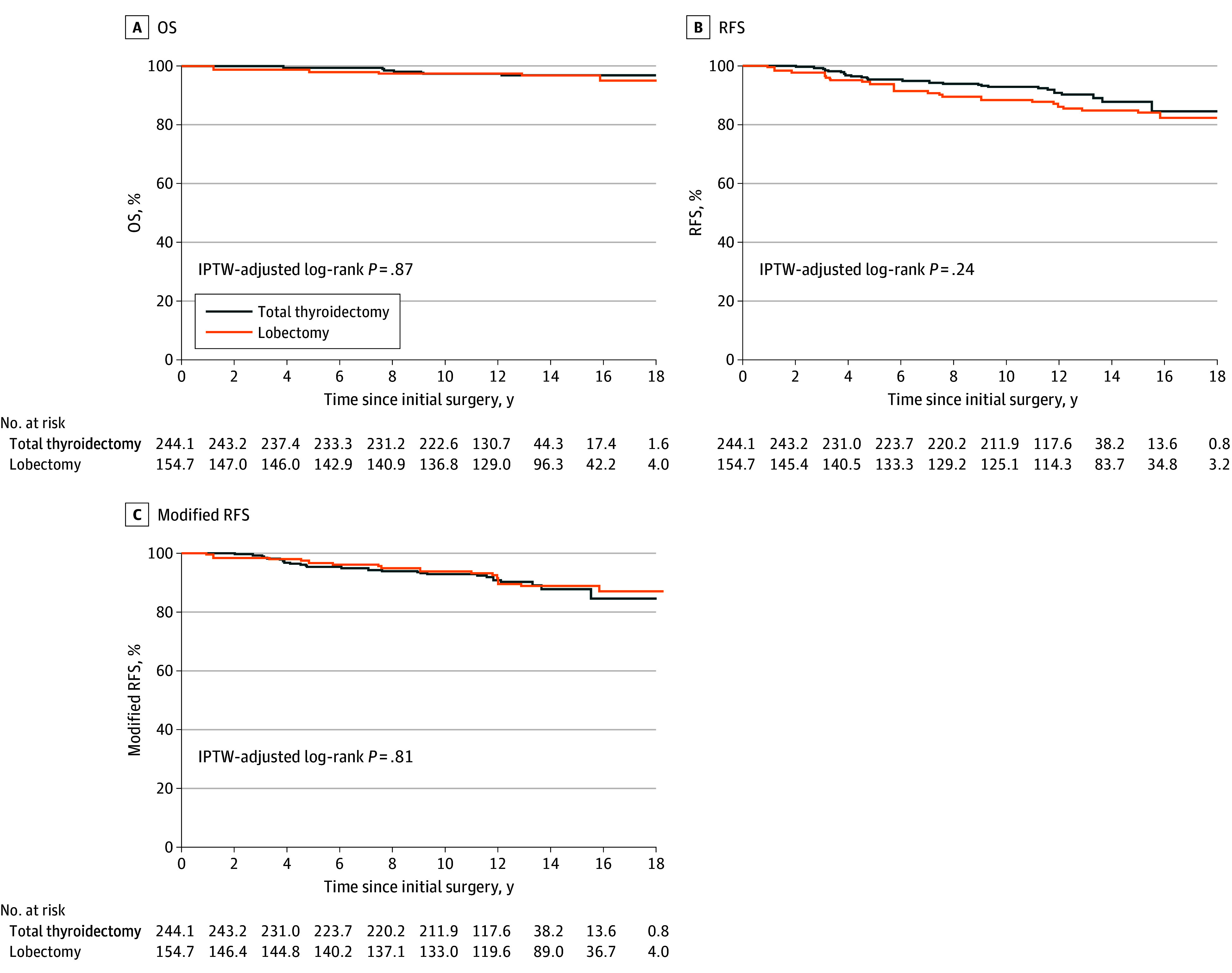
Kaplan-Meier Estimates of Overall Survival (OS), Recurrence-Free Survival (RFS), and Modified RFS After the Inverse Probability of Treatment Weighting (IPTW) analysis In this modified RFS analysis, recurrences limited to the extent that could have been excised if the initial surgery was a total thyroidectomy (ie, remaining thyroid and/or contralateral level VI lymph node) were not counted as events, but recurrences that occurred after a complementary total thyroidectomy and central neck dissection were counted as events. The number at risk reflects the weighted number of patients based on the IPTW method, which may be noninteger values.

The median (IQR) follow-up time was 13.0 (11.2-15.0) years. Figure 3 depicts the patients’ IPTW-adjusted Kaplan-Meier curves, comparing the patients who underwent a lobectomy plus ipsilateral LND with the patients who underwent a total thyroidectomy plus ipsilateral LND. Twelve patients died during the follow-up, and among them, 1 patient died from PTC recurrence (having chosen not to pursue additional treatment after the recurrence). The remaining deaths were attributed to other causes: 4 from other cancers, 2 from cerebrovascular events, 1 from pneumonia, 1 from liver failure, 1 from an accident, 1 from suicide, and 1 from sudden death.

The analysis of the patients' OS detected no clinically meaningful difference between the OS rates of the lobectomy and total thyroidectomy groups. The IPTW-adjusted OS rates at 5, 10, and 15 years were 98.0% (95% CI, 93.9-99.3), 97.5% (95% CI, 93.2-99.1), and 96.8% (95% CI, 92.2-98.7), respectively, for the lobectomy group vs 99.4% (95% CI, 97.0-99.9), 97.4% (95% CI, 94.4-98.8), and 96.9% (95% CI, 93.3-98.5), respectively, in the total thyroidectomy group (hazard ratio [HR], 1.10; 95% CI, 0.35-3.47).

The difference in the RFS values of the lobectomy and total thyroidectomy groups was small and not clinically meaningful. The IPTW-adjusted RFS rates at 5, 10, and 15 years were 93.8% (95% CI, 88.5-96.7), 88.4% (95% CI, 82.0-92.6), and 84.1% (95% CI, 76.8-89.3), respectively, for the lobectomy group vs 95.4% (95% CI, 91.8-97.4), 92.9% (95% CI, 88.8-95.5), and 87.8% (95% CI, 80.8-92.4), respectively, for total thyroidectomy (HR, 1.41; 95% CI, 0.79-2.54). Regarding modified RFS, no clinically meaningful difference was observed between the lobectomy and total thyroidectomy groups. The IPTW-adjusted modified RFS rates at 5, 10, and 15 years were 96.7% (95% CI, 92.2-98.6), 93.8% (95% CI, 88.5-96.7), and 88.9% (95% CI, 82.4-93.1), respectively, for the lobectomy group vs 95.4% (95% CI, 91.8-97.4), 92.9% (95% CI, 88.8-95.5), and 87.8% (95% CI, 80.8-92.4), respectively, for total thyroidectomy (HR, 0.93; 95% CI, 0.49-1.76). The IPTW-adjusted total rate of recurrence during the follow-up was 11.4% (17.6 of 154.7) in patients who underwent lobectomy vs 7.8% (19.1 of 244.1) in patients who underwent total thyroidectomy. The IPTW-adjusted rate of recurrence isolated to the contralateral thyroid or the level VI lymph node area was 4.3% (6.6 of 154.7) in the lobectomy group. Therefore, considering the recurrence location, the IPTW-adjusted rate of recurrence, excluding the contralateral thyroid and the level VI lymph node area, was 7.1% (11.0 of 154.7) in the lobectomy group vs 7.8% (19.1 of 244.1) in the total thyroidectomy group. Detailed information and management of recurrences are provided in the eResults in Supplement 1.

A subgroup analysis was also performed to compare lobectomy and total thyroidectomy without RAI. Similarly, after IPTW adjustment, no clinically meaningful differences between the lobectomy and total thyroidectomy without RAI groups were identified regarding the patients’ 5-year, 10-year, and 15-year OS (98.5% [95% CI, 94.6-99.6], 98.0% [95% CI, 94.0-99.4], and 97.3% [95% CI, 92.9-99.0], respectively, vs 98.9% [95% CI, 93.7-99.8], 98.3% [95% CI, 93.1-99.6], and 97.4% [95% CI, 90.7-99.3]; HR, 1.19; 95% CI, 0.25-5.77), RFS (94.8% [95% CI, 89.8-97.4], 90.2% [95% CI, 84.1-94.0], and 85.6% [95% CI, 78.4-90.6], respectively, vs 96.0% [95% CI, 90.1-98.4], 93.9% [95% CI, 87.4-97.1], and 88.5% [95% CI, 77.3-94.4]; HR, 1.34; 95% CI, 0.63-2.88), or modified RFS (97.1% [95% CI, 92.7-98.8], 94.5% [95% CI, 89.3-97.2], and 90.0% [95% CI, 83.7-93.9], respectively, vs 96.0% [95% CI, 90.1-98.4], 93.9% [95% CI, 87.4-97.1], and 88.5% [95% CI, 77.3-94.4]; HR, 0.90; 95% CI, 0.40-2.02) (eFigure 2 in Supplement 1).

## Discussion

Our analyses revealed no evidence of the superiority of a total thyroidectomy plus ipsilateral LND over a lobectomy plus ipsilateral LND in terms of OS, RFS, or modified RFS in approximately 400 patients with intermediate-risk cN1b PTC with confirmed primary tumors and lymph node metastases in the ipsilateral neck region only. The debate surrounding the optimal surgical management of PTC, particularly in cases with clinically apparent lateral neck lymph node metastasis, remains a topic of interest among clinicians. Our findings contribute to this discourse by shedding light on the long-term survival and recurrence outcomes associated with these 2 surgical approaches.

The results of this study demonstrate that the 2 surgical approaches exhibited overlapping survival curves in patients with PTC limited to the unilateral thyroid lobe and ipsilateral lateral neck lymph nodes. Our findings do not demonstrate statistical inferiority of either surgical approach, and from a clinical perspective, it appears that there is no meaningful difference between the 2 approaches in terms of prognosis and recurrence. However, it is essential to note that lobectomy plus ipsilateral LND cannot prevent recurrence in the area of the remaining thyroid and/or contralateral level VI lymph node. The IPTW-adjusted Kaplan-Meier curves for RFS suggest that lobectomies may have provided a slightly inferior outcome compared with the total thyroidectomies, as the former approach’s curve appears slightly lower; the imprecision in the estimates prevents making definitive conclusions. In contrast, the curves for modified RFS in lobectomy and total thyroidectomy appear to overlap. This observation suggests that lobectomy plus ipsilateral LND cannot prevent recurrence within remaining thyroid and/or contralateral level VI lymph node(s) only, which occurred in approximately 4% of those patients.

A 2023 report from China suggested no significant difference in short-term outcomes between the 2 surgical approaches within a median follow-up of less than 5 years.^21^ Our present investigation, with a median (IQR) follow-up of 13.0 (11.2-15.0) years, further supports these findings by demonstrating favorable treatment outcomes over a long-term period. Given the absence of a meaningful difference in terms of prognosis and recurrence between lobectomy and total thyroidectomy, lobectomy is a viable option. This approach avoids the need for lifelong levothyroxine supplementation, reduces the risk of complications related to the bilateral RLNs and parathyroid glands, and incurs lower medical costs.^7,16,22,23^ Moreover, among all 401 cases in our series, there was only 1 disease-specific death. This result suggests a need to consider the risk of overtreatment in patients designated as intermediate risk.

It is important to acknowledge the ambiguity surrounding the efficacy of RAI therapy for intermediate-risk PTC.^10,11,12^ Our present findings do not provide a basis for establishing lobectomy plus LND as the standard treatment. Intermediate-risk PTC represents a heterogeneous group of carcinomas that encompasses all cases falling outside the categories of low-risk and high-risk PTCs. Among the various patterns within intermediate-risk PTC, cases that remain confined to the unilateral thyroid lobe and ipsilateral lateral neck lymph nodes can be considered relatively closer to the low-risk category. Thus, similar to low-risk PTC, this subgroup of intermediate-risk PTC may exhibit characteristics for which RAI therapy has limited effectiveness. At this point in time, it is therefore reasonable to consider lobectomy plus LND as an equally effective treatment approach in this patient subgroup, alongside total thyroidectomy plus LND.

In addition to the uncertainty surrounding the utility of RAI therapy for intermediate-risk PTC, variations in recommendations across different countries’ guidelines^9,14,15^ further complicates the establishment of a universal standard for lobectomy plus LND in cases such as those examined in this study. Nevertheless, specific scenarios for which lobectomy plus LND may be considered include: (1) patients with intraoperative RLN paralysis resulting in the decision to perform a lobectomy plus ipsilateral LND; (2) patients unable to tolerate long-term levothyroxine, vitamin D, and calcium supplementation; and (3) those preferring to minimize the risk of complications and preserve thyroid function. IONM was not introduced at our hospital during the present study period, but in contemporary procedures, we routinely use IONM for such cases. IONM can help identify intraoperative unilateral RLN paralysis, often leading to the decision to avoid contralateral procedures. Postoperative options may include staged surgery after vocal cord recovery^24,25^ or RAI therapy (ie, lobe ablation)^26,27^ if the vocal cord does not recover, but these add physical and financial burdens on patients. Given our present findings, which did not demonstrate a clear advantage of total thyroidectomy, observation following a lobectomy plus ipsilateral LND is a valid option. Patients who are unable to access long-term supplementation due to personal or financial constraints, such as living in certain countries or regions where obtaining a regular prescription might be difficult, may also find a lobectomy plus LND can be a favorable treatment choice.^28^ For those prioritizing the minimization of complications and the preservation of thyroid function, avoiding a prophylactic contralateral thyroidectomy may reduce risks, although it could increase the likelihood of later structural recurrence in the unaddressed area. Patients accepting this risk may prefer a lobectomy plus ipsilateral LND.

While the above considerations highlight the potential benefits of lobectomy, it is important to recognize the established advantages of total thyroidectomy. A 2023 study indicated that even for high-risk groups, the extent of a thyroidectomy does not necessarily improve prognosis.^29^ However, the significance of total thyroidectomy for PTC might lie more in optimizing the subsequent patient management rather than directly improving the prognosis, especially in patients with high-risk PTC. This significance includes dynamic risk stratification based on thyroglobulin measurements and a determination of the appropriateness of drug therapy based on the patient’s RAI responsiveness. In this context, even in intermediate-risk PTC cases, the role of total thyroidectomy becomes crucial for enabling these postoperative assessments and tailored treatments.

### Limitations

This study has limitations. This was a retrospective analysis with a single-center design. The concept of modified RFS was applied. The treatment groups (lobectomy and total thyroidectomy) were not determined through randomization. Although we used IPTW to balance the patient characteristics, unmeasured confounding factors, such as patients’ adherence to treatment plans, could still influence the outcomes.

The study was conducted at a single thyroid-specific hospital, and the results may not be fully representative of other populations or health care settings. Variations in surgical practices, patient demographic characteristics, and tumor characteristics could have influenced the effects of the surgical strategy on cancer progression and recurrence. Lastly, the concept of modified RFS, which excluded recurrences that could have been excised with a total thyroidectomy, is an assumption that may not fully reflect the clinical reality; this concept is based on the idea that a therapeutic contralateral lobectomy may be required in the future, which introduces a level of uncertainty. While the data used in this study were collected more than 10 years ago, this approach can better reflect the true outcomes of this slow-growing cancer.

## Conclusions

This retrospective cohort study did not detect any superiority of total thyroidectomy plus ipsilateral LND over lobectomy plus ipsilateral LND in terms of patient prognosis or recurrence. Our findings support the idea that individualized treatment decisions, guided by the patients’ characteristics, background, and preferences can lead to favorable outcomes for specific intermediate-risk PTC cases. We hope this research will help inform future guideline revisions and support joint decision-making between patients and their clinicians while improving the overall care and outcomes of patients with intermediate-risk PTC. As the landscape of thyroid cancer management evolves, further research and discussions are warranted to refine the guidelines and tailor treatment strategies to the unique needs of each patient.

## Notes

Supplement 1.
eMethods.

eResults.

eReferences.
**eTable.** Details of Complications Associated With Total Thyroidectomy Before IPTW **eFigure 1.** Kaplan-Meier Estimates of (A) Overall Survival (OS) and (B) Recurrence-Free Survival (RFS) in Patients Undergoing Total Thyroidectomy + Lateral Neck Dissection (LND) Only vs Those Undergoing Total Thyroidectomy + LND Followed by Radioactive Iodine (RAI) Treatment **eFigure 2.** Kaplan-Meier Estimates of (A) OS, (B) RFS, and (C) Modified RFS After IPTW in Patients Undergoing Lobectomy + LND vs Those Undergoing Total Thyroidectomy + LND Without RAI

Supplement 2. Data Sharing Statement
