# Supplementary material for: Lobectomy vs Total Thyroidectomy With Ipsilateral Lateral Neck Dissection for N1b Intermediate-Risk Papillary Thyroid Carcinoma
Source: JAMA Otolaryngol Head Neck Surg. 2024 Nov 27;151(2):105–12. doi: 10.1001/jamaoto.2024.3860 (PMC11826362; doi:10.1001/jamaoto.2024.3860)
Supplement: Supplement 1. — eMethods. eResults. eReferences. eTable. Details of Complications Associated With Total Thyroidectomy Before IPTW eFigure 1. Kaplan-Meier Estimates of (A) Overall Survival (OS) and (B) Recurrence-Free Survival (RFS) in Patients Undergoing Total Thyroidectomy + Lateral Neck Dissection (LND) Only vs Those Undergoing Total Thyroidectomy + LND Followed by Radioactive Iodine (RAI) Treatment eFigure 2. Kaplan-Meier Estimates of (A) OS, (B) RFS, and (C) Modified RFS After IPTW in Patients Undergoing Lobectomy + LND vs Those Undergoing Total Thyroidectomy + LND Without RAI [file jamaotolaryngolheadnecksurg-e243860-s001.pdf]

Supplemental Online Content

Saito Y, Matsuzu K, Abdelhamid Ahmed AH, et al. Lobectomy vs total thyroidectomy with ipsilateral lateral neck dissection for N1b intermediate-risk papillary thyroid carcinoma. *JAMA Otolaryngol Head Neck Surg*. Published online November 27, 2024. doi:10.1001/jamaoto.2024.3860

eMethods.
eResults.
eReferences.
eTable. Details of Complications Associated With Total Thyroidectomy Before IPTW
eFigure 1. Kaplan-Meier Estimates of (A) Overall Survival (OS) and (B) Recurrence-Free Survival (RFS) in Patients Undergoing Total Thyroidectomy + Lateral Neck Dissection (LND) Only vs Those Undergoing Total Thyroidectomy + LND Followed by Radioactive Iodine (RAI) Treatment
eFigure 2. Kaplan-Meier Estimates of (A) OS, (B) RFS, and (C) Modified RFS After IPTW in Patients Undergoing Lobectomy + LND vs Those Undergoing Total Thyroidectomy + LND Without RAI

This supplementary material has been provided by the authors to give readers additional information about their work.

© 2024 Saito Y et al. *JAMA Otolaryngology—Head and Neck Surgery*.

eMethods.

Defining modified recurrence-free survival (RFS)

The modified RFS value applied in this study does not count recurrences at the remaining thyroid or contralateral level VI lymph node as events if the initial surgery was a lobectomy, as these are regions that could have been removed if the initial surgery had been a total thyroidectomy. Specifically, if a recurrence was limited to the remaining thyroid or the contralateral level VI lymph node only, it was not counted as an event in the modified RFS analysis. However, if a recurrence occurred in the remaining thyroid or the contralateral level VI lymph node plus any other sites, it was counted as an event. Additionally, if a recurrence was limited to the remaining thyroid or the contralateral level VI lymph node only, but there was a subsequent recurrence (re-recurrence) after a completion total thyroidectomy and central neck dissection, the re-recurrence was counted as an event. The modified RFS is thus a tailored outcome measure, commonly used and highly effective in clinical research. For example, in studies aiming to assess distant metastasis alone, "distant-recurrence-free survival" or "distant metastasis-free survival" has often been employed, focusing specifically on recurrences at distant sites.[1] Similarly, other modified-RFS parameters have been used to be adapted to focus on conditions such as lymph node recurrences[2] or other site recurrences.[3] In our study, we applied this established methodology to examine recurrences outside of the remaining thyroid tissue.

© 2024 Saito Y et al. *JAMA Otolaryngology—Head and Neck Surgery.*

eResults.

Pathological and intraoperative findings

Before the IPTW adjustment, among the 244 patients in the total thyroidectomy group, 70 (28.7%) had intrathyroidal metastasis in the contralateral lobe, and 58 (23.8%) had metastasis to the contralateral level VI lymph nodes. These were not diagnosed preoperatively but were identified pathologically. The occurrence of aggressive subtypes was low, with only one case of tall cell variant in the total thyroidectomy group.

After the IPTW adjustment, the respective median number of lymph nodes resected in levels II, III, and IV was 21.5 in the lobectomy group and 24.1 in the total thyroidectomy group. The rate of gross extrathyroidal extension was 4.04% in the lobectomy group and 6.19% in the total thyroidectomy group. The rate of patients with more than five pathologic lymph node metastases was 64.0% in the lobectomy group and 73.4% in the total thyroidectomy group, with the total thyroidectomy group also counting metastases to the contralateral level VI lymph nodes.

Recurrence sites

The true recurrence rate, excluding recurrences in the residual thyroid or contralateral level VI, was approx. 7% in each group. After the IPTW adjustment, distant metastasis occurred in 0.57% of the patients in the lobectomy group and 1.49% in the total thyroidectomy group, with all cases involving lung metastases. Recurrence within the

© 2024 Saito Y et al. *JAMA Otolaryngology—Head and Neck Surgery.*

dissected lymph node area was observed in 5.03% of the lobectomy patients and 3.42% of the total thyroidectomy patients. Lymph node recurrence outside the dissected area occurred in 1.53% of the lobectomy patients and 2.61% of those who underwent a total thyroidectomy. Lastly, local neck recurrence was absent in the lobectomy group but occurred in 0.32% of the total thyroidectomy group.

Management after recurrence in the lobectomy group

Before the IPTW adjustment, 17 patients in the lobectomy group experienced recurrence. Among them, 13 patients underwent a completion thyroidectomy during subsequent surgery, one patient had only lymph node dissection, and the remaining three patients were managed with observation alone, with stable recurrent lesions. Of the patients who underwent a completion thyroidectomy after recurrence, only five received additional RAI therapy.

© 2024 Saito Y et al. *JAMA Otolaryngology—Head and Neck Surgery.*

eReferences.

1. Yee D, DeMichele AM, Yau C, et al. Association of event-free and distant recurrence–free survival with individual-level pathologic complete response in neoadjuvant treatment of stages 2 and 3 breast cancer: Three-year follow-up analysis for the I-SPY2 adaptively randomized clinical trial. *JAMA Oncol* 2020;6:1355–1362.

2. Wang LY, Palmer FL, Nixon IJ, et al. Central lymph node characteristics predictive of outcome in patients with differentiated thyroid cancer. *Thyroid* 2014;24:1790–1795.

3. Wiltshire KL, Ward IG, Swallow C, et al. Preoperative radiation with concurrent chemotherapy for resectable rectal cancer: Effect of dose escalation on pathologic complete response, local recurrence-free survival, disease-free survival, and overall survival. *Int J Radiat Oncol Biol Phys* 2006;64:709–716.

© 2024 Saito Y et al. *JAMA Otolaryngology—Head and Neck Surgery.*

eTable. Details of Complications Associated With Total Thyroidectomy Before IPTW

	Lobectomy n=157	Total thyroidectomy n=244
Postoperative hypoparathyroidism:		
Transient	0 (0)	153 (62.7)
Permanent	0 (0)	23 (9.4)
Postoperative bilateral RLN paralysis:		
Transient	0 (0)	2 (0.8)
Permanent	0 (0)	0 (0)

Data are number (percentage). Neither intraoperative neuromonitoring nor near-infrared autofluorescence devices were used during the study period. IPTW: inverse probability of treatment weighting, RLN: recurrent laryngeal nerve.

© 2024 Saito Y et al. *JAMA Otolaryngology—Head and Neck Surgery.*

eFigure 1. Kaplan-Meier Estimates of (A) Overall Survival (OS) and (B) Recurrence-Free Survival (RFS) in Patients Undergoing Total Thyroidectomy + Lateral Neck Dissection (LND) Only vs Those Undergoing Total Thyroidectomy + LND Followed by Radioactive Iodine (RAI) Treatment

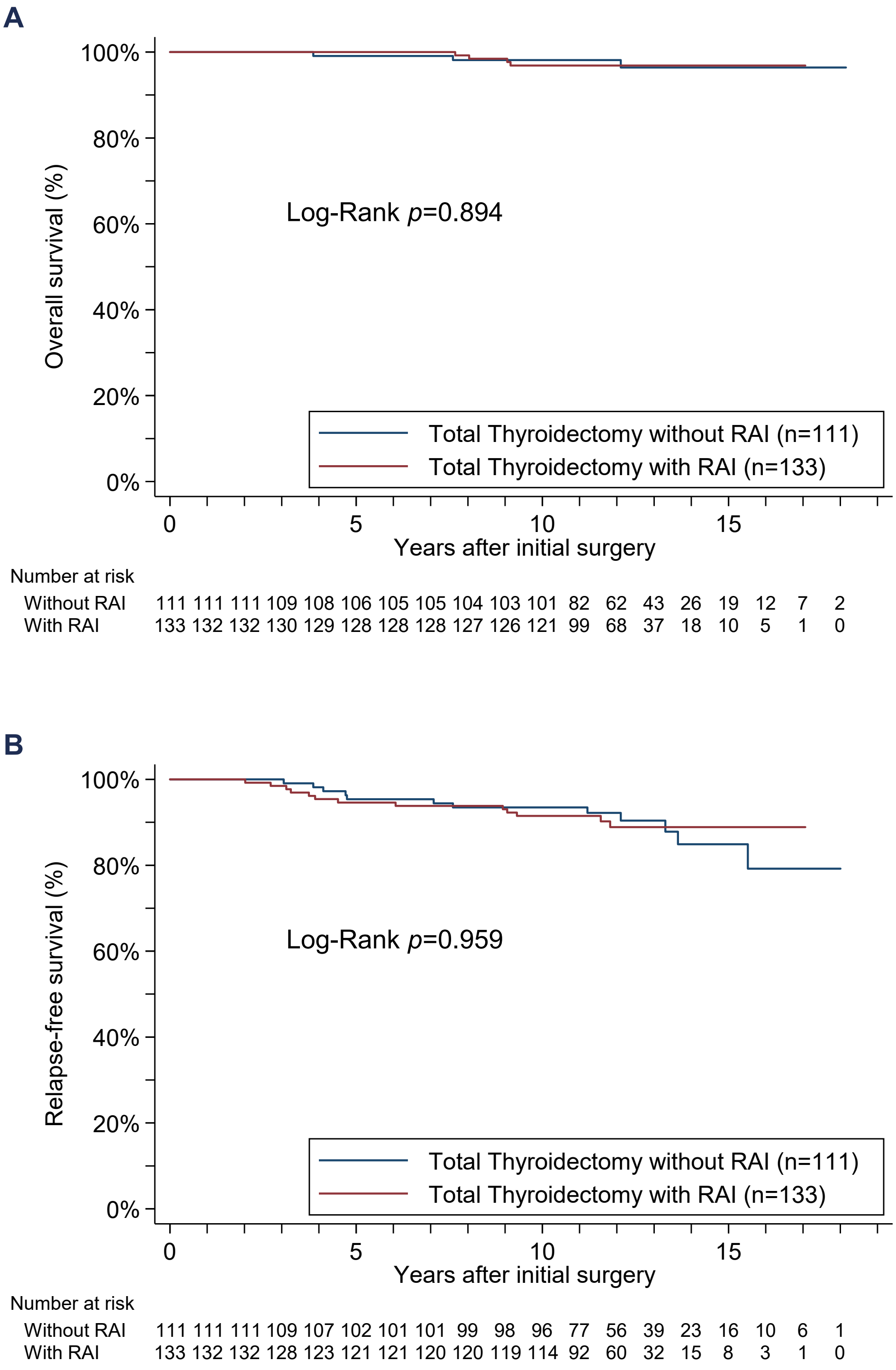

© 2024 Saito Y et al. *JAMA Otolaryngology—Head and Neck Surgery.*

eFigure 2. Kaplan-Meier Estimates of (A) OS, (B) RFS, and (C) Modified RFS After IPTW in Patients Undergoing Lobectomy + LND vs Those Undergoing Total Thyroidectomy + LND Without RAI

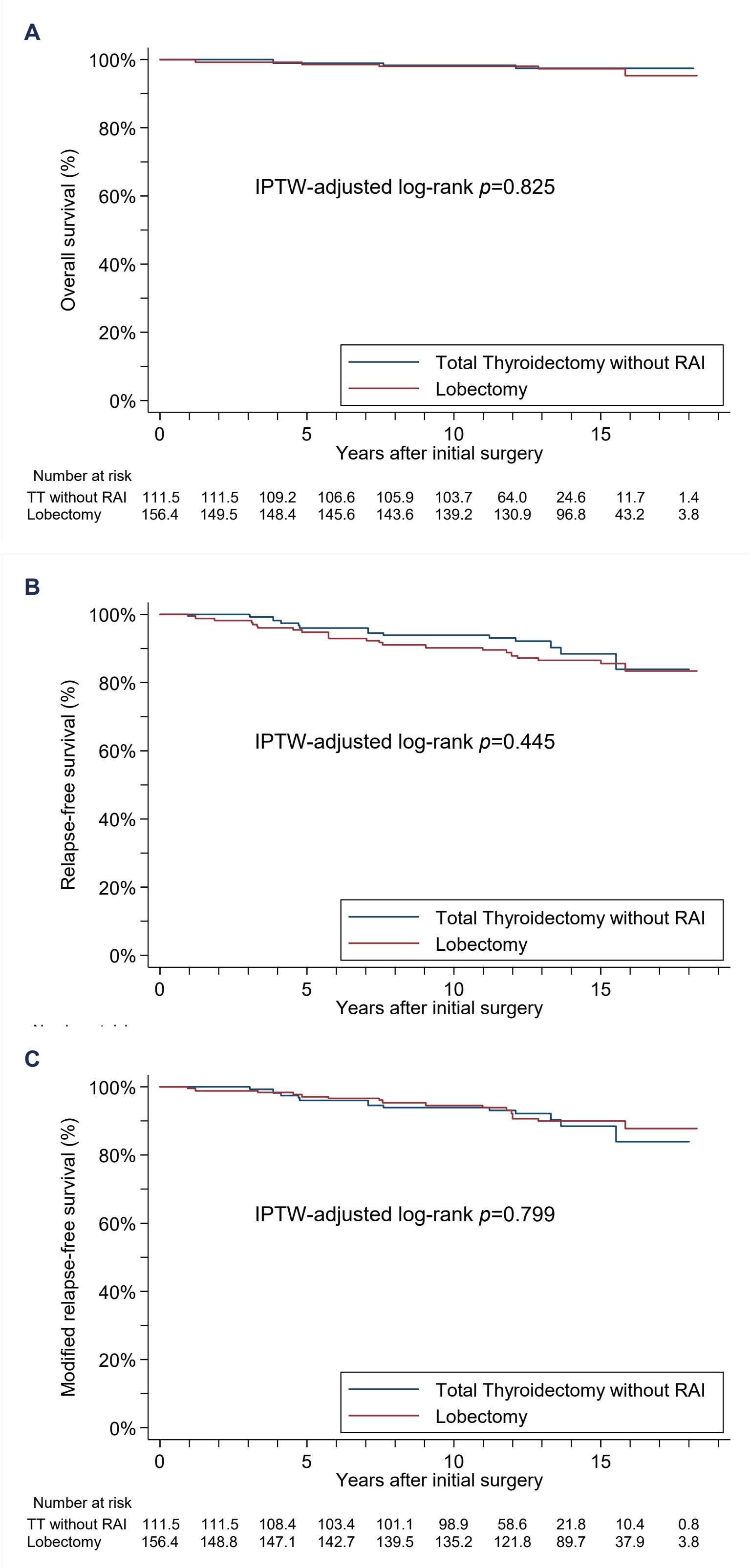

© 2024 Saito Y et al. *JAMA Otolaryngology—Head and Neck Surgery.*
