# Supplementary material for: Lobectomy vs Total Thyroidectomy With Ipsilateral Lateral Neck Dissection for N1b Intermediate-Risk Papillary Thyroid Carcinoma
Source: JAMA Otolaryngol Head Neck Surg. 2024 Nov 27;151(2):105–12. doi: 10.1001/jamaoto.2024.3860 (PMC11826362; doi:10.1001/jamaoto.2024.3860)
Supplement: Supplement 2. — Data Sharing Statement [file jamaotolaryngolheadnecksurg-e243860-s002.pdf]

Data Sharing Statement

Saito. Lobectomy vs Total Thyroidectomy With Ipsilateral Lateral Neck Dissection for N1b Intermediate-Risk Papillary Thyroid Carcinoma. *JAMA Otolaryngol Head Neck Surg*. Published November 27, 2024. doi:10.1001/jamaoto.2024.3860

Data
Data available: Yes
Data types: Deidentified participant data
How to access data: yoshiyuksaito@gmail.com
When available: With publication

Supporting Documents
Document types: Statistical/analytic code
How to access documents: yoshiyuksaito@gmail.com
When available: With publication

Additional Information
Who can access the data: Researchers whose proposed use of the data has been approved
Types of analyses: For any purpose
Mechanisms of data availability: After approval of a proposal
